# Intravenous Iron Therapy in Heart Failure With Iron Deficiency: An Umbrella Review of Systematic Reviews and Meta-Analyses

**DOI:** 10.7759/cureus.114912

**Published:** 2026-08-21

**Authors:** Abhishek Hanumanpratap Singh Kshatri, Samia Arif, Sadia Yousaf, Muhammad Habib, Minahil Saif, Muhammad Muaz Mushtaq

**Affiliations:** 1 Medicine, Employees’ State Insurance Corporation Medical College and Postgraduate Institute of Medical Sciences and Research, Kalaburagi, IND; 2 Medicine, Maharajah's Institute of Medical Sciences, Vizianagaram, IND; 3 Emergency Medicine, Apollo Hospitals Health City, Visakhapatnam, IND; 4 Cardiology, King Edward Medical University, Lahore, PAK; 5 Medicine, King Edward Medical University, Lahore, PAK; 6 Internal Medicine, King Edward Medical University, Lahore, PAK; 7 Medicine and Surgery, King Edward Medical University, Lahore, PAK

**Keywords:** ferric carboxymaltose, ferric derisomaltose, heart failure, iron deficiency, iron repletion

## Abstract

Iron deficiency is a distinct comorbidity in patients with heart failure (HF), independent of anemia, and is associated with reduced functional capacity and increased hospitalization and mortality. Randomized trials of intravenous (IV) iron repletion have yielded directionally consistent but incompletely aligned results, prompting numerous systematic reviews and meta-analyses that largely draw on the same underlying trials. We performed an umbrella review to consolidate this evidence base and clarify the strength and consistency of treatment effects across outcomes. Across the pooled reviews, IV iron, predominantly ferric carboxymaltose (FCM), reduced the composite risk of HF hospitalization and cardiovascular death in patients with heart failure with reduced ejection fraction (HFrEF), an effect attributable chiefly to fewer hospitalizations rather than a survival benefit, which remained non-significant throughout. Functional capacity, symptom class, and quality of life improved consistently. Safety appeared favorable among the outcomes formally assessed, though fewer than half of the included reviews performed a dedicated statistical safety analysis, and known formulation-specific risks such as hypophosphatemia were not evaluated in this literature. The treatment effect appeared attenuated in the one review restricted to acute heart failure, and possible differences in benefit by sex and by baseline iron-transport capacity emerged as hypothesis-generating candidate explanations for residual heterogeneity, warranting prospective confirmation rather than immediate clinical application. Evidence was drawn predominantly from FCM trials, with more limited supporting data for ferric derisomaltose and minimal data for other formulations; findings should not be extrapolated beyond HFrEF, as HF with preserved ejection fraction populations were not separately represented in this literature. Because the available reviews draw heavily on a shared and aging set of trials (corrected covered area of 31.9%, indicating very high primary-study overlap), this synthesis should be regarded as clarifying rather than expanding the evidence base. Taken together, IV iron repletion, particularly with FCM, in iron-deficient HFrEF reduces hospitalizations and improves quality of life, but a mortality benefit has not been established, underscoring the need for adequately powered trials designed specifically to resolve this question.

## Introduction and background

Iron deficiency (ID) is present in up to half of all patients with heart failure (HF), irrespective of anemia status, and is now recognized as a distinct, treatable comorbidity rather than a laboratory abnormality [1]. Iron plays a central role not only in oxygen transport but also in cellular energy metabolism within cardiomyocytes and skeletal muscle, and its deficiency has been independently associated with reduced functional capacity, poorer quality of life, and higher rates of hospitalization and mortality in both acute and chronic HF populations [1,2]. Clinically, this is compounded by the distinction between absolute ID (depleted total body iron stores) and functional ID (adequate stores rendered inaccessible by hepcidin-mediated sequestration under the chronic inflammatory state of HF), a distinction reflected in the ferritin- and transferrin saturation-based diagnostic criteria adopted across major guidelines [1,3,4].

This pathophysiological reasoning motivated a succession of randomized trials (RCTs) of intravenous (IV) iron repletion. Anker et al. demonstrated early symptomatic and functional benefit with ferric carboxymaltose (FCM) in chronic HF, a finding subsequently corroborated in a second trial of similar design by the same group [5,6]. Ponikowski and colleagues extended this work, first confirming improvements in exercise capacity and symptoms in ambulatory chronic HF, and later reporting a reduction in heart failure hospitalizations (HHFs), without significant mortality benefit, in patients with ID assessed shortly after an acute HF admission [7,8]. More recently, Kalra et al. evaluated a different IV iron formulation against standard care in chronic HFrEF, and Mentz et al. conducted the largest cardiovascular (CV) outcomes trial in this space to date; both narrowly missed statistical significance on their prespecified composite primary endpoints, with effect estimates that reached significance only under sensitivity analyses accounting for pandemic-related disruption to follow-up [4,9]. Reflecting this cumulative but imperfectly consistent evidence, the 2021 European Society of Cardiology guidelines assign IV iron a Class IIa recommendation for symptom improvement and hospitalization reduction in iron-deficient HFrEF, and the 2022 American College of Cardiology/American Heart Association guidelines offer comparable endorsement; neither body extends this recommendation to mortality reduction or to the acute HF setting, where evidence remains comparatively sparse [3,10].

The resulting literature is therefore large, mechanistically well-grounded, and clinically consequential, but also heterogeneous. The trials differ in population (ambulatory versus recently hospitalized), iron formulation, ID diagnostic thresholds, and endpoint hierarchy, and no single trial has definitively resolved the question of mortality benefit. This heterogeneity has, in turn, generated a growing number of systematic reviews and meta-analyses (SRMAs), each pooling overlapping subsets of the same primary trials under differing statistical models, subgroup stratifications, and outcome time horizons, and arriving at conclusions that are directionally consistent for hospitalization-related endpoints but discordant in emphasis regarding mortality and acute HF applicability. Because overlap in primary-trial inclusion across these SRMAs risks each being read as independent corroborating evidence when it is not, an umbrella review is warranted to systematically appraise the methodological quality of this secondary literature, quantify the degree of primary-study overlap, and delineate where the totality of evidence supports firm clinical inference versus where uncertainty genuinely persists. Accordingly, this umbrella review was conducted to consolidate the existing SRMA evidence on IV iron therapy in HF with ID and to clarify the strength and consistency of its effects across outcomes and prespecified subgroups.

## Review

Materials and methods

Study Selection

This umbrella review was conducted in accordance with the Preferred Reporting Items for Systematic Reviews and Meta-Analyses (PRISMA) 2020 guidelines and the Preferred Reporting Items for Overviews of Reviews (PRIOR) statement. A comprehensive literature search was performed to identify SRMAs evaluating IV iron therapy in patients with HF and ID. The search was designed to capture quantitative syntheses of RCTs and, where included by the reviews themselves, observational studies examining IV iron use in this population. PubMed, Embase, Scopus, and the Cochrane Library were systematically searched from inception until June 30, 2026. The search strategy combined Medical Subject Headings (MeSH) and free-text keywords such as "intravenous iron," "ferric carboxymaltose," "ferric derisomaltose," "iron deficiency," and "heart failure." No language restrictions were applied. Reference lists of all included articles were manually screened to identify additional eligible reviews. Search results were imported into Rayyan for deduplication, followed by screening. Two reviewers (S.A. and M.S.) independently assessed titles and abstracts to determine potential eligibility, and the same reviewers retrieved full-text versions of potentially relevant articles for detailed evaluation. Disagreements regarding inclusion were resolved through discussion and, if necessary, arbitration by a third reviewer (M.H.). The selection process was documented using a PRISMA flow diagram outlining the number of records identified, screened, excluded, and ultimately included in the final analysis. This umbrella review was not registered on PROSPERO or an equivalent registry. The complete search strategies for all databases, including the exact search terms and Boolean operators used, are presented in Appendices A, B, C, and D.

Eligibility Criteria

Eligible studies included systematic reviews or meta-analyses that examined the use of IV iron therapy in adult patients with HF (acute or chronic, across the spectrum of ejection fraction) and a diagnosis of ID, defined per each review's own criteria. To be considered, reviews must have included at least one RCT involving human participants aged 18 years or older; reviews incorporating a minority of observational studies alongside a predominantly RCT-based evidence base were also eligible. IV iron exposure was defined broadly, including any formulation (e.g., FCM, ferric derisomaltose), dose, or regimen. Reviews comparing IV iron to placebo or standard of care without iron repletion were considered eligible. Only reviews that explicitly evaluated clinical outcomes such as HHF, CV or all-cause mortality, functional capacity, quality of life, or safety endpoints were included. Reviews that did not report outcomes specific to IV iron separately from other interventions, narrative reviews, scoping reviews, conference abstracts, and reviews lacking a systematic methodology or a clearly defined literature search were excluded. Articles focused solely on preclinical, animal, or in vitro studies were also excluded, as was gray literature. The complete inclusion and exclusion criteria, with each criterion presented separately, are provided in Table 1.

**Table 1:** Inclusion and exclusion criteria ID, iron deficiency; RCT, randomized trial; IV, intravenous; HF, heart failure; FCM, ferric carboxymaltose; HHF, heart failure hospitalization; CV, cardiovascular

Category	Criterion
Inclusion	Adult patients (≥18 years) with HF (acute or chronic, across the spectrum of ejection fraction) and a diagnosis of ID, defined per each review's own criteria
Inclusion	Systematic review or meta-analysis including at least one randomized controlled trial with human participants
Inclusion	Reviews incorporating a minority of observational studies alongside a predominantly RCT-based evidence base
Inclusion	IV iron therapy of any formulation (e.g., FCM, ferric derisomaltose), dose, or regimen
Inclusion	Comparator of placebo or standard of care without iron repletion
Inclusion	Explicit reporting of clinical outcomes specific to IV iron (HHF, CV or all-cause mortality, functional capacity, quality of life, or safety endpoints), reported separately from other interventions
Exclusion	Reviews that did not report outcomes specific to IV iron separately from other HF interventions
Exclusion	Narrative reviews
Exclusion	Scoping reviews
Exclusion	Conference abstracts
Exclusion	Reviews lacking a systematic methodology or a clearly defined literature search
Exclusion	Studies focused solely on preclinical, animal, or in vitro data
Exclusion	Gray literature

Data Extraction

Data were independently extracted from each included review by two reviewers (S.Y. and A.N.) using a pre-designed standardized extraction form. Variables included first author, year of publication, number and design of included primary studies, total sample size and allocation between intervention and control arms, population characteristics (acute vs. chronic HF, ejection fraction category), ID diagnostic definition used, and reported primary and secondary outcomes. For reviews reporting pooled effect estimates, extracted data also included odds ratios, risk ratios, mean differences, 95% confidence intervals, p-values, I² values for heterogeneity, and details of subgroup, sensitivity, or meta-regression analyses where performed. Any discrepancies in data extraction were resolved through discussion or with input from a third reviewer (M.H.).

Quality Assessment

The methodological quality of all included SRMAs was assessed using the A Measurement Tool to Assess Systematic Reviews (AMSTAR 2) checklist [11]. This tool evaluates systematic reviews across 16 domains, including protocol registration, comprehensiveness of the literature search, duplicate study selection and data extraction, risk-of-bias assessment of primary studies, appropriateness of meta-analytic methods, accounting for risk of bias in result interpretation, and assessment of publication bias. Each review was independently rated by two reviewers (M.H. and S.Y.) as high, moderate, low, or critically low confidence based on the presence or absence of critical and non-critical flaws according to AMSTAR 2 guidance. Disagreements in scoring were resolved through discussion and consensus. Reviews with no critical weaknesses and only one non-critical weakness were rated high confidence; those with no critical weaknesses but more than one non-critical weakness were rated moderate confidence; those with one critical weakness were rated low confidence; and those with more than one critical weakness were rated critically low confidence. An explicit "No" response on a critical item was treated as a weakness; an "Unclear" response, reflecting incomplete reporting by the original review rather than a confirmed methodological flaw, was not by itself treated as disqualifying.

Data Analysis

Given that this umbrella review synthesized previously published SRMAs with substantial overlap in their constituent primary studies, a de novo meta-analysis was not conducted. Instead, a narrative synthesis of findings across reviews was performed, focusing on the direction, magnitude, and consistency of treatment effects for HHF, mortality, functional/quality-of-life outcomes, and safety, contextualized alongside heterogeneity estimates (I²), GRADE certainty ratings where reported, and AMSTAR 2 confidence ratings. Given the recurrence of several frequently cited primary trials across the 15 included reviews, the degree of primary-study overlap was formally quantified using the corrected covered area (CCA) statistic [12]. It is calculated as CCA=(N−r)/(r×c−r), where N is the total number of primary-study citations across all reviews, r is the number of unique primary randomized controlled trials, and c is the number of included reviews. A citation matrix was constructed by cross-referencing the reference list of each included review against the pooled set of unique primary trials, with observational studies excluded from the primary CCA denominator given their inconsistent inclusion across reviews. Overlap was interpreted according to Pieper's classification (slight, 0-5%; moderate, 6-10%; high, 11-15%; very high, >15%), and pairwise CCA values between individual review pairs were additionally calculated to characterize the distribution of overlap across the review set.

Results

Study Selection Process

**Figure 1:**
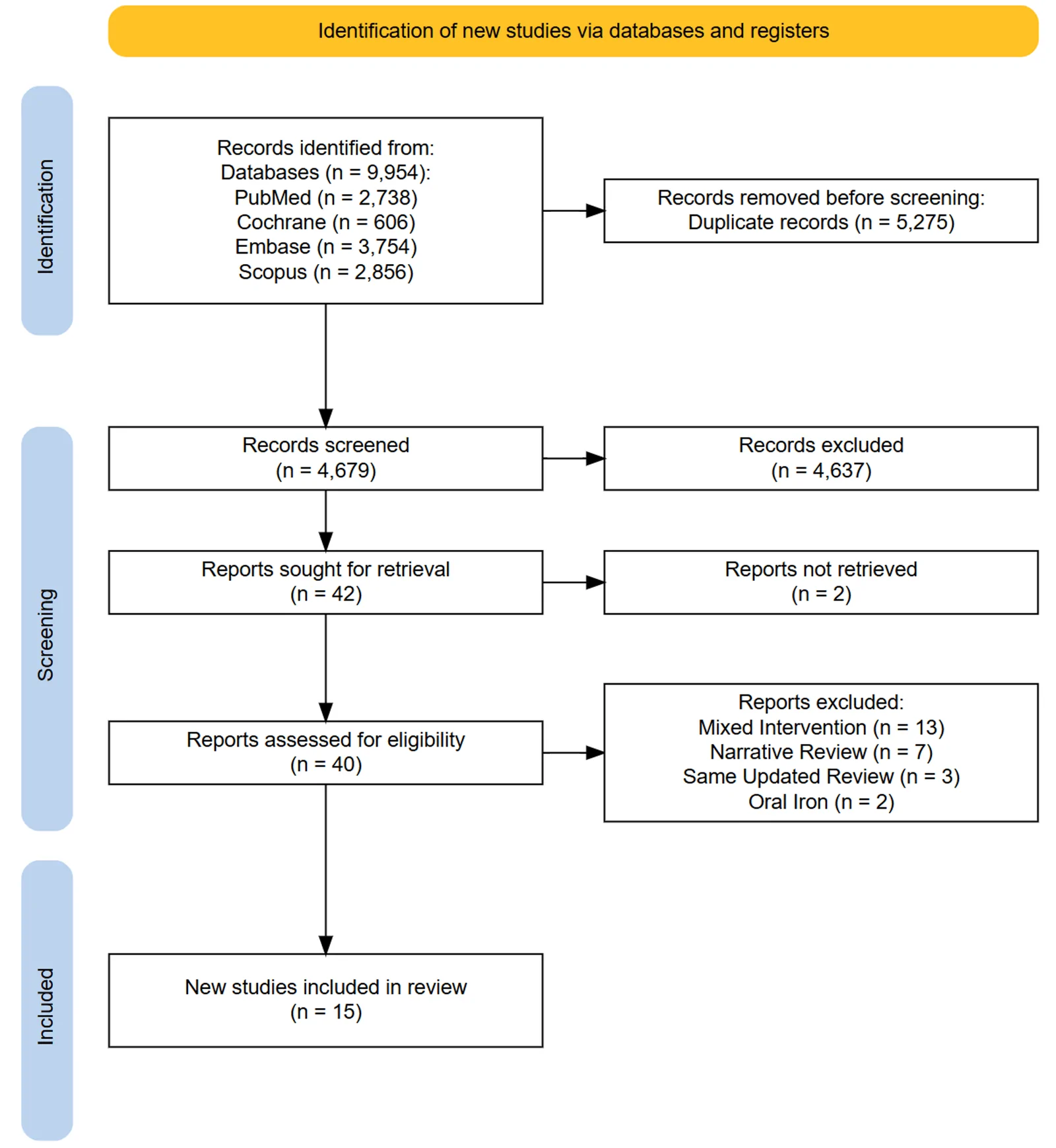
PRISMA diagram illustrating the study selection process PRISMA, Preferred Reporting Items for Systematic Reviews and Meta-Analyses

A total of 9,954 records were initially retrieved through systematic searches across PubMed, Embase, Scopus, and the Cochrane Library. After the removal of 5,275 duplicate entries, 4,679 unique records remained for screening. Following the initial screening phase, 42 studies were identified as potentially eligible and were retrieved for full-text evaluation. Upon thorough assessment against the predefined inclusion and exclusion criteria, 15 SRMAs met all eligibility requirements and were included in the final umbrella review [2,6,7,12-23]. The remaining 25 full-text articles were excluded for various reasons, including reviews that did not report IV iron-specific outcomes separately from other HF interventions (n=13), reviews that were superseded by or fully overlapped with a more recent, updated meta-analysis from the same research group (n=3), narrative reviews lacking a systematic methodology or clearly defined search strategy (n=7), and studies addressing oral iron supplementation rather than IV iron (n=2). No additional studies were identified through manual screening of the reference lists of the included reviews or related publications. The entire selection process is summarized in the PRISMA flow diagram (Figure 1).

Study Characteristics

Fifteen SRMAs, published between 2023 and 2026, were included in this umbrella review [2,6,7,13-24]. Collectively, these reviews synthesized data from a combined pool of three to 18 primary studies each, encompassing a total patient population ranging from 3,008 to 7,813 across the individual reviews. The primary studies underpinning these reviews were predominantly randomized controlled trials; only two reviews incorporated a mix of randomized and observational evidence, Lopez et al. (3 RCTs and 6 observational studies) and Taha et al. (10 RCTs and 3 observational/cohort studies), while the remaining thirteen reviews relied exclusively on RCT-derived evidence [19,24].

The included populations across all 15 reviews consisted of adult patients with HF and biochemically confirmed ID, most commonly enrolling both acute and chronic HF cohorts, while four reviews restricted enrollment to acute HF or HF with reduced ejection fraction (HFrEF) specifically [15,16,19,22]. The intervention under investigation was consistently IV iron therapy, predominantly FCM, compared against heterogeneous control regimens across reviews, including placebo, standard of care, or no iron administration (Table 2). Four of the 15 included reviews restricted their pooled analysis to FCM exclusively [7,13,22,24]. The remaining eleven incorporated at least one additional formulation, most commonly ferric derisomaltose or iron sucrose and ferric gluconate from smaller, older trials. Two reviews were nominally framed around FCM specifically, based on their titles, but on inspection of their included trial sets incorporated non-FCM formulations, representing an internal inconsistency between stated scope and actual pooled composition that limits the interpretability of their formulation-specific claims [15,23].

**Table 2:** Study characteristics of included studies 6-MWT, 6-minute walk test; AEs, adverse events; AHF, acute heart failure; CV, cardiovascular; eGFR, estimated glomerular filtration rate; EQ-5D, EuroQol-5 Dimension; VAS, visual analog scale; FCM, ferric carboxymaltose; GI, gastrointestinal; Hb, hemoglobin; HF, heart failure; HFrEF, heart failure with reduced ejection fraction; HHFs, heart failure hospitalization(s); HR, hazard ratio; ID, iron deficiency; IV, intravenous; KCCQ, Kansas City Cardiomyopathy Questionnaire; LVEF, left ventricular ejection fraction; MLHFQ, Minnesota Living with Heart Failure Questionnaire; NYHA, New York Heart Association; QoL, quality of life; RRR, relative risk reduction; TSA, trial sequential analysis; TSAT, transferrin saturation; VO₂, oxygen consumption (peak VO₂, peak oxygen consumption); RCT, randomized trial

Author	Year	Studies included	Sample size	Population	ID definition used	Formulation(s) studied	Primary outcomes	Secondary outcomes
Ahmed et al. [13]	2026	11 RCTs	6493 (3329 FCM; 3164 control)	Both AHF and chronic HF	Ferritin <100 ng/mL; or ferritin 100-299 ng/mL with TSAT <20%, per most trials' criteria	FCM only	Composite of recurrent HHF or CV mortality, assessed at 1 year and complete follow-up	Recurrent HHF (alone), all-cause mortality, CV mortality, 6-MWT, AEs
Anker et al. [6]	2025	6 RCTs	7175 (3672 IV iron; 3503 control)	Both AHF and chronic HF	Serum ferritin <100 µg/L, or serum ferritin 100-299 µg/L with TSAT <20% (all trials used this definition; IRONMAN excluded ferritin ≥400 µg/L, others excluded ≥300 µg/L)	Mixed: FCM (5); ferric derisomaltose (1)	Composite of recurrent HHFs (total events) and CV mortality, assessed (i) within 12 months and (ii) over complete follow-up	Recurrent HHFs alone; CV mortality alone; all-cause mortality; safety endpoints (infections, serious AEs); subgroup analyses (sex, age, ischemic vs. non-ischemic etiology, NYHA class, eGFR, Hb, ferritin, TSAT)
Lopez et al. [19]	2025	9 (3 RCTs, 6 observational studies)	3588 (1759 IV iron; 1936 control)	AHF only	Ferritin <100 μg/L; or ferritin 100-299/300 μg/L+TSAT <20%; or TSAT <20% alone (thresholds varied slightly by study)	Mixed: predominantly FCM (81.9% of IV-iron patients); iron sucrose; iron dextran; sodium ferric gluconate complex	HHF; all-cause rehospitalization; all-cause mortality; mean change in Hb level	Not explicitly differentiated as primary vs. secondary; study treated all four outcomes as co-primary outcomes of interest
Shalabi et al. [23]	2025	9 RCTs	7491 (3829 IV iron; 3662 control)	Both AHF and chronic HF	Serum ferritin <100 μg/L, or ferritin 100-300 μg/L with TSAT <20% (used in 8/9 trials); Dhoot et al. included ID patients without reporting specific diagnostic thresholds	Titled as FCM-specific; includes ferric derisomaltose (1) among 9 trials	First HHF or CV mortality (composite)	Total HHFs; first HHF (alone); CV mortality (alone); total all-cause hospitalization; NYHA class improvement; patient global assessment improvement
Mhanna et al. [20]	2024	14 RCTs	6614 (3394 IV iron; 3220 control)	Both AHF and chronic HF	Serum ferritin <100 ng/mL, or 100-300 ng/mL with TSAT ≤20%	Mixed: predominantly FCM; iron sucrose (3); ferric derisomaltose (1); ferric gluconate (1)	Composite of HHFs or CV-related mortality; HHFs; all-cause mortality; HF-related mortality; CV-related mortality	NYHA functional classification improvement; QoL (validated questionnaires); 6-MWT; LVEF; AEs (GI side effects, infections, vascular complications)
Awad et al. [15]	2024	18 RCTs	7813 (3998 IV iron; 3815 control)	Both AHF and chronic HF; all HFrEF	Not explicitly stated	Framed as “IV FCM”; actual set mixed iron sucrose (5); ferric gluconate (1); ferric derisomaltose (2); FCM (10)	Mean change from baseline in KCCQ, NYHA class, serum ferritin, TSAT, LVEF%, 6-MWT, Hb, peak VO₂	All-cause hospital admission, all-cause mortality, mortality due to worsening HF, CV hospitalization
Bhatia et al. [16]	2024	9 RCTs	3337 (1756 IV iron; 1581 control)	Both AHF and chronic HF; all HFrEF	Mostly ferritin <100 μg/L, or ferritin 100-300 μg/L with TSAT <20%	Predominantly FCM; iron sucrose (2); ferric gluconate (1); ferric derisomaltose (1)	Composite of first HHF or CV mortality	HHF (first or recurrent), CV mortality, all-cause mortality, composite of any-cause hospitalization or mortality, GI side effects, any infection; subgroup analyses (sex, HF etiology, NYHA class, TSAT); meta-regression (baseline Hb); TSA
Martens et al. [18]	2024	14 RCTs	6624 (3407 IV iron; 3217 control)	Both AHF and chronic HF	Ferritin <100 ng/mL, or ferritin 100-300 ng/mL if TSAT <20% (most common definition across trials); some trials used ferritin <100 or TSAT <20% alone	Mixed: FCM (9); iron sucrose (2, one combined with FCM); ferric derisomaltose (1); ferric gluconate (1)	CV mortality; CV mortality+HHF (combined); first HHF; total HHFs	Meta-regression by baseline TSAT to explain heterogeneity; sensitivity analyses using random-effects model, HR-based pooling for time-to-event data, and stratification by follow-up duration (≤24 vs. >24 weeks); treatment effect modification by iron formulation (FCM vs. non-FCM)
Taha et al. [24]	2024	13 studies (10 RCTs, 3 observational/cohort studies)	6271 (3260 IV iron; 3011 control)	Both AHF and chronic HF	Hb <7.7 mmol/L (13 g/dL) in men and <7.4 mmol/L (12 g/dL) in women, along with HF	FCM only	6-MWT distance; EQ-5D VAS	KCCQ; difference in Hb; all-cause mortality; all-cause mortality at 1 year; first HHF or CV mortality; first HHF during follow-up; CV mortality; CV mortality or intervention for worsening HF during follow-up
Rangwala et al. [22]	2024	7 RCTs	5229 (2691 IV iron; 2538 control)	Both AHF and chronic HF; all HFrEF	Not explicitly stated	FCM only	CV mortality; all-cause mortality; composite of CV mortality or HHF; HHF	At least one serious AE; subjects experiencing serious cardiac events; treatment discontinuation events; Hb levels; injection site reactions
Graham et al. [17]	2023	10 RCTs	3373 (1759 IV iron; 1614 control)	Both AHF and chronic HF	Varied by trial but most commonly: serum ferritin <100 μg/L regardless of TSAT, or ferritin 100-300 μg/L with TSAT <20%. IRONMAN allowed TSAT <20% if ferritin <400 μg/L	Mixed: FCM (7 trials); ferric derisomaltose (1); iron sucrose (2)	Composite of recurrent HHF and CV mortality	Composite of first HHF or CV mortality; recurrent HHF alone; CV mortality; all-cause mortality (each also analyzed at 1-year follow-up)
Oli et al. [21]	2023	16 RCTs	6732 (3463 IV iron; 3269 control)	Both AHF and chronic HF	Not explicitly stated	Mixed: FCM (10 studies); iron sucrose (3); ferric derisomaltose (2); sodium ferric gluconate complex (1)	Composite of HHF or CV mortality; all-cause hospitalizations; hospitalization for worsening HF; all-cause mortality; CV mortality; mortality due to worsening HF	Functional outcomes: change in 6-MWT, LVEF, peak VO₂, KCCQ, MLHFQ. Safety outcomes: total AEs, serious AEs, premature discontinuation of study medication
Anker et al. [14]	2023	4 RCTs	3008 (1581 IV iron; 1427 control)	Both AHF and chronic HF	Not explicitly stated	Mixed: FCM (3); ferric derisomaltose (1)	Composite of total (first and recurrent) HHFs and CV mortality	Subgroup interaction effects (RRR) for: sex, age (<69.4 vs. ≥69.4 y), HF etiology (ischemic vs. non-ischemic), TSAT (<20% vs. ≥20%), eGFR (≤60 vs. >60 mL/min/1.73m²), Hb (<11.8 vs. ≥11.8 g/dL), ferritin (<35 vs. ≥35 μg/L), NYHA class (II vs. III/IV)
Salah et al. [2]	2023	10 RCTs	3438 (1801 IV iron; 1637 control)	Both AHF and chronic HF	Generally defined as serum ferritin <100 μg/L, or ferritin 100-300 μg/L if TSAT <20% (slight variation across RCTs)	Mixed: FCM (6); iron sucrose (2); ferric derisomaltose (1); ferric gluconate (1)	Composite of CV mortality and first HHF; all-cause mortality; CV mortality; first HHF; total HHFs	None specified separately (all listed outcomes were treated as co-primary/prespecified outcomes of interest)
Ponikowski et al. [7]	2023	3 RCTs	4501 (2251 IV iron; 2250 control)	Both AHF and chronic HF	Same definition across all 3 FCM trials: ferritin <100 ng/mL, or ferritin 100-300 ng/mL with TSAT <20%	FCM only; ferric derisomaltose (1) as separate aggregate-data sensitivity analysis only	Co-primary endpoint 1: composite of total/recurrent CV hospitalizations and CV mortality. Co-primary endpoint 2: composite of total HHFs and CV mortality (both through 52 weeks)	(i) Time to first CV hospitalization or CV mortality; (ii) time to first HHF or CV mortality; (iii) rate of total HHFs; (iv) time to first HHF; (v) time to CV mortality; (vi) time to all-cause mortality; (vii) total CV hospitalizations; (viii) time to first CV hospitalization; (ix) total all-cause hospitalization

The primary outcome assessed across most of the 15 included reviews was a composite of HHF (recurrent or first-event) and CV mortality, though the specific formulation varied in terms of timing (e.g., 12 months vs. complete follow-up) and event counting (total vs. first event) across reviews. Secondary outcomes varied considerably but commonly included all-cause mortality, functional capacity (6-minute walk test), quality of life (Kansas City Cardiomyopathy Questionnaire (KCCQ), EuroQol-5 Dimension (EQ-5D) visual analog scale (VAS)), New York Heart Association (NYHA) functional class, hemoglobin and ferritin change, and safety endpoints including adverse events and infections. Awad et al. and Taha et al. prioritized functional and quality-of-life measures as their primary outcomes rather than the composite hospitalization/mortality endpoint used elsewhere [15,24]. The main findings of the included studies are summarized in Table 3.

**Table 3:** Summary of the main findings and conclusions of included studies 6-MWT, 6-minute walk test; ACC, American College of Cardiology; AHA, American Heart Association; HFSA, Heart Failure Society of America; AEs, adverse events; AHF, acute heart failure; CI, confidence interval; CV, cardiovascular; eGFR, estimated glomerular filtration rate; EQ-5D, EuroQol-5 Dimension; VAS, visual analogue scale; ESC, European Society of Cardiology; FCM, ferric carboxymaltose; GI, gastrointestinal; GRADE, Grading of Recommendations Assessment, Development and Evaluation; Hb, hemoglobin; HF, heart failure; HFrEF, heart failure with reduced ejection fraction; HHFs, heart failure hospitalizations; HR, hazard ratio; I², I-squared (statistical heterogeneity); ID, iron deficiency; IPD, individual patient data; IV, intravenous; KCCQ, Kansas City Cardiomyopathy Questionnaire; LVEF, left ventricular ejection fraction; MD, mean difference; MLHFQ, Minnesota Living with Heart Failure Questionnaire; NNT, number needed to treat; NYHA, New York Heart Association; OR, odds ratio; QoL, quality of life; RR, risk ratio; RRR, relative risk reduction; SMD, standardized mean difference; TSA, trial sequential analysis; TSAT, transferrin saturation; VO₂, oxygen consumption (peak VO₂, peak oxygen consumption)

Author	Main findings	Conclusions
Ahmed et al. [13]	FCM significantly reduced recurrent HHF or CV mortality at 1 year (RR 0.73, 95% CI 0.62-0.85) and complete follow-up (RR 0.80, 95% CI 0.68-0.94); recurrent HHF alone was also reduced at 1 year (RR 0.69) and complete follow-up (RR 0.75); trends toward reduced all-cause mortality (RR 0.86) and CV mortality (RR 0.86) at 1 year attenuated at complete follow-up (RR 0.95 and 0.90, respectively); 6-MWT significantly improved (MD 29.19 m, 95% CI 11.95-46.43); no significant increase in AEs (RR 1.02); TSA confirmed robust evidence for the primary composite outcome	IV FCM in HF patients with ID was associated with a reduced risk of adverse CV events (recurrent HHF/CV mortality) and improved functional capacity (6-MWT); mortality benefit showed only a non-significant trend that attenuated with longer follow-up; further trials are needed to clarify the long-term survival benefit
Anker et al. [6]	IV iron reduced the primary composite endpoint at 12 months (RR 0.72, 95% CI 0.55-0.89) and over complete follow-up (RR 0.81, 95% CI 0.63-0.97). Recurrent HHFs were reduced at 12 months (RR 0.69, 95% CI 0.48-0.88) and over complete follow-up (RR 0.78, 95% CI 0.55-0.98). CV mortality showed a non-significant trend toward reduction at 12 months (HR 0.80, 95% CI 0.61-1.03) and over complete follow-up (HR 0.87, 95% CI 0.73-1.04). All-cause mortality similarly showed a non-significant trend toward reduction (HR 0.82, 95% CI 0.65-1.03 at 12 months; HR 0.92, 95% CI 0.80-1.07 over complete follow-up). No safety signal was observed for infection (OR 1.02, 95% CI 0.66-1.59) or serious AEs (OR 0.91, 95% CI 0.70-1.15). A significant treatment interaction was observed by sex (RRR 1.40, 95% CI 1.05-1.86), with no benefit in women (RR 0.98, 95% CI 0.75-1.26) and greater benefit in men. No significant interactions were observed by age, etiology, NYHA class, eGFR, Hb, ferritin, or TSAT	Treating ID with IV iron in patients with HFrEF significantly reduces the composite of recurrent HHFs and CV mortality, driven mainly by hospitalization reduction. Effects were greatest in the first year post-randomization and were consistent across most subgroups, including baseline TSAT. Whether women derive less benefit requires further study. Overall safety was confirmed, with no excess infection or AE signal
Lopez et al. [19]	HHF: RR 0.96 (95% CI 0.76-1.21; P=0.74; I²=74%). All-cause rehospitalization: RR 1.03 (95% CI 0.90-1.19; P=0.64; I²=3%). All-cause mortality: RR 1.00 (95% CI 0.81-1.24; P=0.87; I²=0%). Hb change: MD 0.80 g/dL (95% CI 0.33-1.27; P=0.0003; I²=88%), a statistically significant increase favoring IV iron. GRADE certainty: low (HHF); moderate (all-cause mortality); moderate (all-cause rehospitalization)	IV iron therapy in AHF with ID significantly increases Hb levels but does not significantly reduce HHF, all-cause rehospitalization, or all-cause mortality. Findings are consistent with the 2021 ESC guidelines, which give IV iron a Class IA recommendation in chronic HF but make no formal recommendation for AHF because of limited evidence. Larger, standardized RCTs with longer follow-up and LVEF-stratified analyses are needed to clarify the clinical benefit
Shalabi et al. [23]	Primary outcome: RR 0.89 (95% CI 0.83-0.96; P=0.0016), I²=0%. Total HHFs: RR 0.82 (95% CI 0.77-0.87; P<0.0001), I²=65.8%. First HHF: RR 0.89 (95% CI 0.80-1.00; P=0.0406 under fixed-effects; not significant under random-effects). CV mortality: RR 0.89 (95% CI 0.80-0.99; P=0.0384), I²=0%. Total all-cause hospitalization: RR 0.93 (95% CI 0.85-1.01; P=0.0830; not significant), I²=62.5%. NYHA improvement: OR 2.75 (95% CI 1.86-4.06; P<0.0001). Patient global assessment: OR 2.40 (95% CI 1.85-3.11; P<0.0001). TSA: conclusive for the primary outcome and total HHFs; suggestive but inconclusive for first HHF, CV mortality, and all-cause hospitalization	IV FCM significantly reduces the composite risk of HHF or CV mortality and total HHFs in patients with ID and HFrEF; it does not significantly reduce all-cause hospitalization. FCM also improves functional status (NYHA class) and patient-reported QoL. Findings support routine ID screening in HFrEF and reinforce FCM as a targeted therapeutic strategy, although limitations include IPD unavailability, dosing heterogeneity across trials, and ongoing debate over the validity of ferritin-based ID definitions
Mhanna et al. [20]	Composite outcome: RR 0.84 (95% CI 0.73-0.96; P=0.01), I²=44%. HHF: RR 0.74 (95% CI 0.61-0.89; P=0.002), I²=62% (sensitivity analysis excluding CONFIRM-HF and Toblli: RR 0.81, 95% CI 0.72-0.91; I²=32%). CV mortality: RR 0.90 (95% CI 0.80-1.00; P=0.06), I²=0%. HF mortality: RR 0.50 (95% CI 0.20-1.23; P=0.13), I²=0%. All-cause mortality: RR 0.90 (95% CI 0.80-1.02; P=0.09), I²=1%. NYHA: MD -0.61 (95% CI -0.97 to -0.24; P=0.001). QoL: SMD 1.9 (95% CI 0.47-3.3; P=0.009). 6-MWT: MD 22.3 m (95% CI 5.99-38.6; P=0.007). LVEF: MD 2.7% (95% CI -0.96 to 6.36; P=0.15), not significant. AEs (GI, infections, vascular): no significant increase	IV iron therapy reduces HHFs and the composite of HHF/CV mortality and improves NYHA class, QoL, and 6-MWT distance, but has no significant effect on LVEF, all-cause mortality, CV mortality, or HF mortality. The safety profile appears favorable, with no increased risk of AEs. Further large-scale RCTs are needed to confirm long-term efficacy and safety
Awad et al. [15]	IV iron significantly improved KCCQ (MD 7.39), LVEF (MD 3.76%), 6-MWT (MD 34.87 m, driven by chronic HF), NYHA class (MD -1.09), Hb (MD 1.44), and serum ferritin (MD 152.65) (all P<0.001). No significant differences were observed in TSAT or peak VO₂. IV iron significantly reduced HHF (RR 0.75), CV hospitalization (RR 0.50), and all-cause mortality (RR 0.80), with no significant difference in all-cause hospitalization or mortality due to worsening HF	IV iron improves Hb, LVEF, QoL (KCCQ), and functional status (NYHA class, 6-MWT, peak VO₂) in acute and chronic HFrEF and reduces HHF, CV hospitalization, and all-cause mortality, supporting its use as standard of care in HF with ID, although the clinical significance of LVEF gains remains uncertain
Bhatia et al. [16]	IV iron reduced the composite of first HHF or CV mortality by 16% (RR 0.84, 95% CI 0.75-0.93; NNT 18), driven mainly by a 25% reduction in HHF (RR 0.75, 95% CI 0.64-0.88; NNT 10). It also reduced the composite of any-cause hospitalization or mortality by 8% (RR 0.92, 95% CI 0.85-0.99; NNT 19). No significant effect was observed on CV mortality (RR 0.89) or all-cause mortality (RR 0.94), with no increase in GI events or infections. No publication bias was detected, and heterogeneity was low throughout. Benefit was greater in ischemic vs. non-ischemic HF (subgroup P=0.036), with no significant interaction by sex, NYHA class, or TSAT. TSA confirmed that the primary and HHF results crossed both statistical and Lan-DeMets sequential boundaries, supporting a true benefit	IV iron reduces HHF/CV mortality, driven by fewer hospitalizations, without increased mortality, infection, or GI risk. Effects were consistent across trials, and TSA confirmed the finding as robust rather than a false positive
Martens et al. [18]	IV iron reduced CV mortality (OR 0.867, 95% CI 0.755-0.955, P=0.0427), combined CV mortality + HHF (OR 0.838, 95% CI 0.751-0.936, P=0.0015), first HHF (OR 0.855, 95% CI 0.744-0.983, P=0.0281), and total HHFs (RR 0.739, 95% CI 0.661-0.827, P<0.0001). Significant heterogeneity was observed for first HHF (I²=60.3%) and total HHFs (I²=75.6%); meta-regression showed greater benefit in trials with lower baseline TSAT (≤20%). No heterogeneity was observed for CV mortality (I²=0%), and no treatment effect modification by iron formulation was identified	IV iron reduces both CV mortality and HF-related events across a broad HF population; baseline TSAT, particularly ≤20%, may be an important determinant of the magnitude of benefit for HF-related events, supporting a call to reconsider/redefine the ID definition in HF
Taha et al. [24]	6-MWT: SMD 1.45 (95% CI 0.55-2.36; P=0.002; I²=99%). EQ-5D VAS: SMD 1.56 (95% CI -0.33-3.45; P=0.11; I²=99%; non-significant). KCCQ: SMD 1.49 (95% CI 0.87-2.11; P<0.00001; I²=98%). First HHF or CV mortality: RR 0.91 (95% CI 0.84-0.98; P=0.02; I²=0%). First HHF during follow-up: RR 0.87 (95% CI 0.69-1.09; P=0.22; I²=61%; non-significant). CV mortality: RR 0.90 (95% CI 0.77-1.05; P=0.17; I²=0%; non-significant). CV mortality or intervention for worsening HF: RR 0.41 (95% CI 0.04-4.51; P=0.47; I²=66%; non-significant). All-cause mortality: RR 0.89 (95% CI 0.69-1.16; P=0.28; I²=0%; non-significant)	FCM significantly improves functional capacity (6-MWT) and QoL (KCCQ) in HF patients with ID anemia but has no significant effect on mortality or hospitalization rates. Findings highlight the need for further research addressing both symptomatic and survival aspects of HF management
Rangwala et al. [22]	No significant effect on CV mortality (OR 0.88; 95% CI 0.72-1.07; P=0.56) or all-cause mortality (OR 0.87; 95% CI 0.72-1.04; P=0.46). Significant reduction in the composite endpoint of CV mortality/HHF (OR 0.60; 95% CI 0.38-0.93; I²=84%; P=0.0001) and HHF alone (OR 0.66; 95% CI 0.48-0.91; I²=54%), with findings robust in sensitivity analysis excluding CONFIRM-HF (OR 0.82; 95% CI 0.71-0.94; I²=0%). No significant difference in serious cardiac events (OR 0.71; 95% CI 0.49-1.03) or treatment discontinuation (OR 0.87; 95% CI 0.67-1.12). Hb was significantly higher with FCM (MD 0.50; 95% CI 0.48-0.52; I²=0%). No significant difference in at least one serious AE overall (OR 0.89; 95% CI 0.65-1.23; I²=78%); however, sensitivity analysis excluding HEART-FID showed fewer events with FCM (OR 0.76; 95% CI 0.62-0.94; I²=0%). Injection-site reactions were more frequent with FCM (OR 2.53; 95% CI 1.15-5.59; I²=0%)	FCM significantly reduces HF-related hospitalizations and the composite of HHF/CV mortality, but does not significantly improve overall or CV mortality in HF patients with ID. Findings support FCM's role in current guidelines as a therapeutic option for correcting ID in HF
Graham et al. [17]	IV iron reduced the composite of recurrent HHF and CV mortality: RR 0.75 (95% CI 0.61-0.93; P<0.01). First HHF or CV mortality: OR 0.72 (95% CI 0.53-0.99; P=0.04). Recurrent HHF alone: RR 0.67 (95% CI 0.47-0.97; P=0.03). CV mortality: OR 0.86 (95% CI 0.70-1.05; P=0.14), inconclusive. All-cause mortality: OR 0.93 (95% CI 0.78-1.12; P=0.47), inconclusive. Subgroup analysis showed a trend toward greater benefit in patients with low TSAT (<20%) (OR 0.67 vs. OR 0.99 for TSAT ≥20%; interaction P=0.07), but this was not statistically significant	In aggregate data from 10 RCTs, IV iron reduced the risk of the composite of first and recurrent HHF or CV mortality, driven primarily by a reduction in HHF. Effects on CV and all-cause mortality remain inconclusive
Oli et al. [21]	IV iron significantly reduced the composite of HHF/CV mortality (OR 0.56, 95% CI 0.40-0.79; I²=83%) and HHF (OR 0.69, 95% CI 0.54-0.90; I²=57%). No significant effect on all-cause hospitalization, all-cause mortality, or CV mortality. Significant improvements in 6-MWT (MD 20.02, 95% CI 8.16-31.87), LVEF (MD 2.03, 95% CI 0.49-3.58), and MLHFQ (MD -8.48, 95% CI -16.20 to -0.76). No significant change in peak VO₂ or KCCQ. No significant differences in total AEs, serious AEs, or premature discontinuation	IV iron therapy is effective in reducing HF-related hospitalization without increasing the risk of serious AEs and improves functional/exercise performance from baseline compared with control (placebo or standard of care). No significant effect was demonstrated on all-cause or CV mortality. Larger trials with longer follow-up are warranted to clarify the effects on mortality
Anker et al. [14]	Overall RR 0.728 (95% CI 0.476-0.992; τ=0.16); prediction interval 0.335-1.378. Individual trial estimates: FAIR-HF, 0.460 (0.177-1.194); CONFIRM-HF, 0.514 (0.277-0.952); AFFIRM-AHF, 0.757 (0.600-0.955); IRONMAN, 0.820 (0.660-1.019). No statistically significant differential treatment effect across any subgroup (all RRR 95% CIs crossed 1.0). Sensitivity analysis (half-normal prior scale 1.0): RR 0.724 (0.24-1.055); τ=0.20	IV iron significantly reduces recurrent HHFs and CV mortality in patients with ID and HFrEF; effects were nominally consistent across subgroups, although subgroup analyses lacked statistical power and results require confirmation in ongoing trials (HEART-FID, FAIR-HF2)
Salah et al. [2]	IV iron significantly reduced the composite of CV mortality + first HHF (RR 0.85, 95% CI 0.77-0.95; I²=0%), first HHF (RR 0.82, 95% CI 0.67-0.99; I²=8%); total HHFs (RR 0.74, 95% CI 0.60-0.91; I²=47%). No significant difference was observed in all-cause mortality (RR 0.95, 95% CI 0.83-1.09; I²=0%) or CV mortality (RR 0.89, 95% CI 0.75-1.05; I²=0%)	IV iron infusion in HF with ID (regardless of anemia) produces a 15% reduction in the composite risk of CV mortality/first HHF (driven mainly by a reduction in hospitalization) and a 26% reduction in total HHFs, with no effect on all-cause or CV mortality alone; findings align with the 2022 ACC/AHA/HFSA and 2021 ESC guideline recommendations for IV iron in HFrEF
Ponikowski et al. [7]	(i) Time to first CV hospitalization or CV mortality; (ii) time to first HHF or CV mortality; (iii) rate of total HHFs; (iv) time to first HHF; (v) time to CV mortality; (vi) time to all-cause mortality; (vii) total CV hospitalizations; (viii) time to first CV hospitalization; (ix) total all-cause hospitalizations	In patients with HF with reduced LVEF and ID, IV FCM significantly reduced the risk of hospitalization for HF and CV causes, with no apparent effect on mortality. The treatment effect was driven by reduced hospitalization rates rather than improved survival. FCM was safe and well tolerated, with no new safety signals. The effect appeared consistent across sex, age, and baseline ferritin, with possible greater benefit in patients with ischemic HF etiology and low baseline TSAT

Quality Assessment

The AMSTAR-2 quality assessment revealed variable methodological rigor across the 15 included studies, with confidence ratings ranging from low to moderate (Table 4). Ten reviews were rated moderate confidence, satisfying the critical domains but showing gaps such as absent or unconfirmed protocol registration, single-database or partial search strategies, unclear or missing excluded-studies documentation, or inability to formally test publication bias due to small numbers of pooled studies [2,13,15-21,24]. Three reviews were rated low confidence, each carrying one confirmed critical weakness, most commonly an explicit absence of publication-bias assessment despite adequate study numbers [6,22,23]. Two reviews were rated critically low confidence because both were individual-patient-data re-analyses of a small, pre-selected set of known trials rather than de novo systematic reviews, and accumulated critical weaknesses across protocol registration, search comprehensiveness, risk-of-bias methodology, and publication-bias assessment [7,14]. Ponikowski et al. was additionally industry-funded and industry-provided the pooled trial data [7]. No review was excluded from the umbrella review on the basis of quality assessment, given that these methodological patterns were considered when interpreting the consistency and strength of the pooled findings reported across reviews rather than being used as an exclusion criterion.

**Table 4:** AMSTAR-2 quality assessment of the 15 included SRMAs evaluating intravenous iron therapy in HF with ID AMSTAR 2, A Measurement Tool to Assess Systematic Reviews; PICO, population, intervention, comparator, outcome; PROSPERO, International Prospective Register of Systematic Reviews; OSF, Open Science Framework; RoB, risk of bias; TSA, trial sequential analysis; GRADE, Grading of Recommendations Assessment, Development and Evaluation; ROBINS; ID, iron deficiency; RCT; HF, heart failure; SRMAs, systematic reviews and meta-analyses

AMSTAR-2 item	Ahmed, 2026 [13]	Anker, 2025 [6]	Lopez, 2025 [19]	Shalabi, 2025 [23]	Mhanna, 2024 [20]	Awad, 2024 [15]	Bhatia, 2024 [16]	Martens, 2024 [18]	Taha, 2024 [24]	Rangwal, 2024 [22]	Graham, 2023 [17]	Oli, 2023 [21]	Anker, 2023 [14]	Salah, 2023 [2]	Ponikowski, 2023 [7]
1. PICO stated	Yes	Yes	Yes	Yes	Yes	Yes	Yes	Yes	Yes	Yes	Yes	Yes	Yes	Yes	Yes
2. Pre-registered protocol	Yes	Yes	Yes	Yes	Unclear	Yes	Unclear	Yes	Unclear	Unclear	Yes	Yes	No	Unclear	No
3. Explanation of study design choice	No	No	No	No	No	No	No	No	No	No	No	No	No	No	No
4. Comprehensive search	Partial yes	Partial yes	Yes	Yes	Yes	Yes	Yes	Partial yes	Yes	Yes	Partial yes	Yes	No	Partial yes	No
5. Duplicate study selection	Yes	Yes	Yes	Yes	Yes	Unclear	Yes	Yes	Yes	Yes	Yes	Yes	No	Yes	Unclear
6. Duplicate data extraction	Yes	Yes	Yes	Yes	Yes	Unclear	Yes	Yes	Yes	Yes	Yes	Yes	Unclear	Yes	Unclear
7. Excluded-studies list with reasons	Unclear	Unclear	Unclear	Unclear	Unclear	Unclear	Unclear	Unclear	Unclear	Unclear	Unclear	Unclear	No	Unclear	Unclear
8. Adequate description of studies	Yes	Yes	Yes	Yes	Yes	Yes	Yes	Yes	Yes	Yes	Yes	Yes	Yes	Yes	Yes
9. Satisfactory RoB technique	Yes (RoB 2)	Yes (RoB, per Cochrane)	Yes (RoB 2+ROBINS-I)	Yes (RoB 2)	Partial yes (Jadad scale)	Yes (RoB 2)	Yes (implied Cochrane RoB)	Yes (RoB 2)	Partial yes (RoB 2 for RCTs only; no ROBINS-I for the 3 observational studies)	Yes (Cochrane RoB)	Yes (RevMan/Cochrane RoB)	Yes (RoB 2.0)	No	Yes (RoB 2)	No
10. Funding of included studies reported	No	No	No	No	No	No	No	No	No	No	Unclear	No	No	No	No
11. Appropriate meta-analytical methods	Yes	Yes	Yes	Yes	Yes	Yes	Yes	Yes	Yes	Yes	Yes	Yes	Yes	Yes	Yes
12. RoB impact on results assessed	Yes (GRADE)	Unclear	Yes (GRADE)	Unclear	No	Unclear	Unclear	Unclear	Unclear	Unclear	Unclear	Unclear	No	Unclear	Unclear
13. RoB in interpretation/discussion	Yes	Unclear	Yes	Unclear	Unclear	Unclear	Unclear	Unclear	Unclear	Unclear	Unclear	Unclear	No	Partial yes	Unclear
14. Heterogeneity explained	Yes	Yes	Yes	Yes	Yes	Yes	Yes	Yes	Yes	Yes	Yes	Yes	Yes	Yes	Yes
15. Publication bias investigated	Unclear (TSA used, not Egger's)	No (too few studies)	Yes	No (too few studies)	Yes (funnel+Egger's)	Unclear	Yes (funnel plots)	Unclear	Unclear	No (too few studies)	Unclear	Yes (funnel plots)	No	Partial yes (limited by high I²)	No
16. Conflicts of interest disclosed	Yes	Yes	Yes	Yes	Yes	Yes	Unclear	Yes	Yes	Unclear	Unclear	Yes	Yes	Unclear	Yes
Overall confidence	Moderate	Low	Moderate	Low	Moderate	Moderate	Moderate	Moderate	Moderate	Low	Moderate	Moderate	Critically low	Moderate	Critically low

Primary Study Overlap

**Figure 2:**
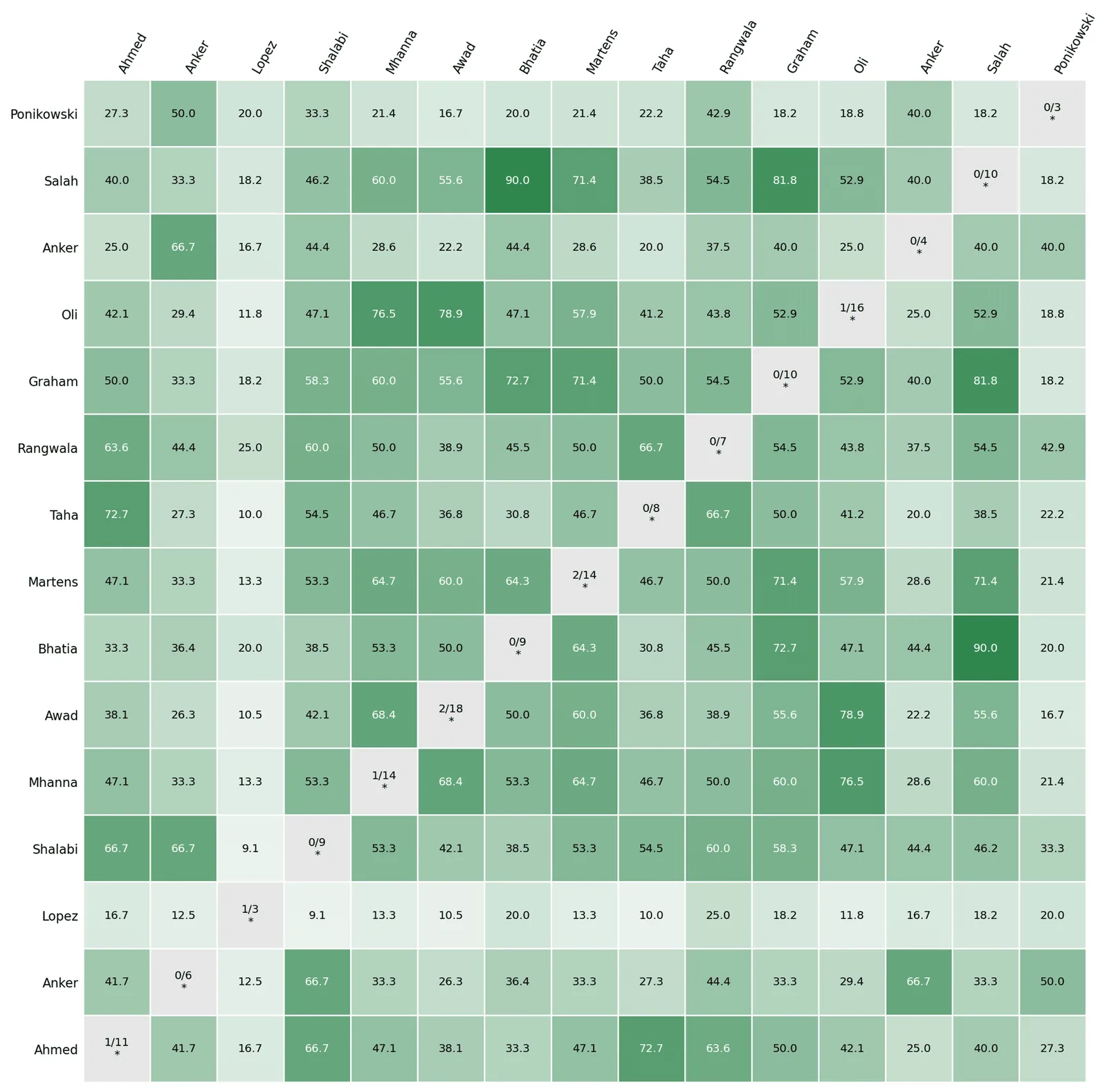
Pairwise CCA between the 15 included SRMAs Darker green shading indicates a higher degree of primary-study overlap between the corresponding pair of reviews (CCA=0%, white, indicates no shared primary trials; CCA=100%, deep green, indicates complete overlap of primary trials). Diagonal values denote the number of primary trials cited by only that review relative to the total number of primary trials included in that review. SRMAs, systematic reviews and meta-analyses; CCA, corrected covered area

Across the 15 included SRMAs, a total of 26 unique primary randomized controlled trials were cited. The overall CCA was 31.9%, corresponding to a very high degree of overlap (Figure 2). A small number of pivotal trials drove this overlap disproportionately. CONFIRM-HF and AFFIRM-HF were each cited by 14 of the 15 reviews, and FAIR-HF by 13, whereas several smaller or more recently published trials were cited by only a single review each [5,8,25]. This very high overall overlap indicates that the apparent convergence of pooled effect estimates across reviews, discussed below, partly reflects shared source data rather than fully independent replication. The full citation matrix is provided in Appendix E.

Discussion

This umbrella review synthesized findings from 15 SRMAs evaluating IV iron therapy in patients with HF and ID. Across the included reviews, a consistent signal emerged: IV iron therapy, predominantly FCM, reduces the composite risk of HHF and CV mortality, with pooled risk ratios ranging from approximately 0.72 to 0.89 in the majority of reviews [2,6,7,13,14,16-18,20,21,23]. However, given the very high primary-trial overlap identified by our CCA analysis (31.9%), this convergence should not be interpreted as independent corroboration; it substantially reflects repeated analysis of the same landmark trials rather than replication across distinct evidence streams. Somewhat more reassuringly, trial sequential analyses performed within several of the constituent reviews indicate that the required information size for the primary composite outcome had already been met, which is a check on statistical robustness that is not undermined by review-level overlap in the same way [13,16,23].

Notably, this benefit appears to be driven predominantly by a reduction in HHFs rather than by improvements in mortality. This pooled hospitalization benefit itself reflects a mix of first-event and recurrent/total-event analyses across reviews; the latter mechanically yield larger relative risk reductions by counting multiple hospitalizations per patient, whereas reviews reporting first hospitalization alone generally showed more modest, though still directionally consistent, effect sizes. Across nearly every review, all-cause and CV mortality showed, at best, a non-significant trend toward benefit that frequently attenuated with longer follow-up or diminished once dosing was discontinued beyond the first year, as highlighted by the inclusion of the FAIR-HF2 trial in the most recent pooled analysis [6,13,17]. This pattern suggests that the clinical value of IV iron lies chiefly in reducing morbidity and healthcare utilization rather than extending survival, a distinction with direct implications for how the therapy is positioned in guideline-directed medical therapy and how its cost-effectiveness should be evaluated [26].

Interpretation by clinical setting is constrained by how the constituent reviews themselves handled population heterogeneity. Eleven of the 15 included reviews pooled acute and chronic HF populations together without reporting acute-versus-chronic subgroup results separately, precluding a stratified synthesis at the umbrella-review level for these reviews. Three reviews restricted their pooled analysis to HFrEF specifically, but likewise did not separate acute from chronic populations within that restriction. The one exception, Lopez et al., restricted its analysis to acute HF exclusively, and found no significant reduction in HHF, all-cause rehospitalization, or mortality, despite a significant increase in hemoglobin [19]. This discordance is instructive rather than contradictory: it suggests that the treatment effect observed in the broader, chronic-predominant literature may be attenuated or absent in the acute post-decompensation setting, where confounding by illness severity and competing risks is greater, though we caution that this inference rests on a single review rather than a stratified analysis across the evidence base. This is consistent with current European guidelines, which extend a stronger recommendation for IV iron specifically in chronic HF while stopping short of a comparable recommendation in the acute setting [3]. We recommend that the global statement that IV iron reduces hospitalization be read as most directly supported for chronic, stable HF populations, with acute HF representing a genuinely distinct and comparatively under-evidenced clinical scenario.

Beyond the primary composite endpoint, functional and patient-reported outcomes showed consistent improvement across reviews that assessed them. Six-minute walk test distance, NYHA functional class, and quality-of-life measures (KCCQ, EQ-5D) improved significantly in several analyses, even where hospitalization or mortality benefits were less robust [13,15,20,21,24]. This reinforces that IV iron's symptomatic and functional benefits are a robust and independent component of its clinical value, distinct from its effects on hard outcomes.

Safety findings were reassuring and consistent; no review identified a significant increase in serious adverse events, infections, or gastrointestinal side effects, aside from a higher rate of injection-site reactions with FCM noted in one review [22]. However, this reassurance should be read against how unevenly safety was assessed across the included evidence base: only five of the 15 reviews performed a dedicated, formally tested statistical analysis of adverse events [6,13,20-22]. Several others reported safety descriptively or indirectly without formal pooled testing, and seven of the 15 reviews reported no adverse event outcome at all. Moreover, none of the 15 included reviews assessed hypophosphatemia or hypersensitivity reactions as distinct safety outcomes, despite hypophosphatemia being a recognized, formulation-specific adverse effect of FCM described in the broader IV iron literature outside HF. We regard the absence of a signal for these outcomes as reflecting absence of assessment rather than confirmed absence of risk, and caution against interpreting the overall safety profile as comprehensively characterized.

Subgroup analyses, where reported, did not identify robust effect modification by age, HF etiology, or NYHA class, though two findings merit specific attention; a formal sex-by-treatment interaction reported in a single review (RRR 1.40, 95% CI 1.05-1.86), with women showing no significant benefit and men benefiting more, and a meta-regression suggesting that baseline transferrin saturation, rather than ferritin alone, may be a more important determinant of treatment response, a finding with potential implications for how ID itself should be defined and operationalized in future trials and clinical practice [6,18]. This association is derived from trial-level meta-regression rather than individual-patient-level data, and is therefore subject to ecological bias: an association between a trial's average baseline TSAT and its average treatment effect does not necessarily reflect a true patient-level treatment-effect interaction. We regard this finding as hypothesis-generating rather than as established evidence of a patient-level effect modifier, pending confirmation in individual-patient-data analyses. Similarly, the sex-by-treatment interaction was identified in only one of the 15 included reviews, was not reported as a prespecified subgroup analysis to our knowledge, and has not been corroborated by any other review; subgroup interaction findings of this kind remain susceptible to reduced statistical power, multiple-testing inflation, and chance, and this result should likewise be regarded as hypothesis-generating rather than as a confirmed treatment-effect modifier that should inform sex-based treatment decisions.

The PICO framework was deliberately defined broadly, spanning the full spectrum of HF etiology, acuity, ejection fraction, and IV iron formulation/regimen, because this reflects how the underlying primary-trial and secondary-review literature has itself been structured. The great majority of published SRMAs in this space, and the RCTs they pool, do not stratify by left ventricular ejection fraction (LVEF) phenotype or by formulation, making a narrower a priori PICO unworkable without excluding most of the eligible evidence base. Where the constituent reviews did restrict their populations (to HFrEF specifically, or to acute HF only), this is reported explicitly. Post hoc stratification by LVEF phenotype (HFmrEF, HFpEF) was not possible, as none of the 15 included reviews reported outcomes separately for these subgroups. Several further methodological considerations temper the strength of these conclusions. First, the definition of ID varied meaningfully across reviews and, more fundamentally, across the primary trials they pooled, most commonly ferritin <100 ng/mL or 100-299/300 ng/mL with TSAT <20%, but with inconsistent thresholds and, in several reviews, no explicit standardization at all [15,21,22]. This heterogeneity in the exposure definition itself introduces uncertainty into pooled effect estimates and complicates cross-review comparison. Second, our AMSTAR-2 appraisal identified substantial overlap in the primary trials underpinning these 15 reviews; the same landmark trials recur across nearly every review, meaning the apparent consistency of findings partly reflects shared source data rather than fully independent replication. Third, quality assessment revealed substantial methodological limitations as no review met criteria for high confidence. Ten reviews were rated moderate confidence, three low confidence, and two critically low confidence. These ratings describe the methodological conduct of the reviews themselves and should not be conflated with the certainty of evidence for individual clinical outcomes, assessed separately via GRADE, nor with the consistency of directionally similar findings across reviews, which, as above, partly reflects shared primary-trial overlap rather than independent replication. Overlap was quantified using CCA (31.9%, "very high"), which confirms and formalizes the qualitative impression from the AMSTAR-2 appraisal that a small set of landmark trials recur across nearly every included review; residual uncertainty remains regarding how this trial-level double-counting propagates into the direction and precision of pooled estimates reported by the constituent reviews themselves.

Consistent with umbrella review methodology, a de novo meta-analysis of primary trial data was not undertaken; conclusions instead rely on the pooled estimates, risk-of-bias judgments, and, where reported, GRADE certainty ratings generated by the constituent reviews themselves, without independent re-abstraction of individual study- or participant-level data. Two of the 15 included reviews incorporated a minority of observational studies alongside randomized evidence, introducing a modest, review-specific risk of confounding not shared by the remaining 13 exclusively RCT-based reviews. We did not exclude these two reviews from our synthesis, but our overall conclusions are weighted toward the concordant findings of the 13 RCT-exclusive reviews, with Lopez et al. and Taha et al. treated as supportive rather than load-bearing evidence [19,24].

Taken together, this umbrella review provides high-level evidence that IV iron, particularly FCM, meaningfully reduces HHFs in patients with chronic HF and ID, with a favorable safety profile and consistent functional benefit, but without a clearly established mortality benefit. This pattern of consistent morbidity benefit alongside uncertain mortality benefit should inform how clinicians counsel patients and how guideline bodies and payers weigh the therapy's value, particularly as cost-effectiveness discussions increasingly hinge on hospitalization avoidance rather than survival gains [23]. Our conclusions most directly and robustly apply to FCM, which comprises the large majority of pooled patients across the constituent reviews. The consistent direction of effect in reviews incorporating ferric derisomaltose provides some, though not comprehensive, support for a broader IV iron class effect. Evidence for iron sucrose and ferric gluconate remains confined to small, older trials with limited pooled weight, and these formulations differ from FCM in iron content, dosing schedule, and infusion protocol; they should not be assumed to be clinically interchangeable with FCM on the basis of this evidence. The variable AMSTAR-2 confidence ratings and substantial primary-trial overlap identified across the included reviews further suggest that future updates to this literature should prioritize transparent overlap quantification and registered protocols, rather than continuing to generate additional overlapping meta-analyses of the same trial pool. Future research should prioritize harmonized ID definitions, dedicated trials in acute HF populations, and further exploration of sex-based and TSAT-based effect modification to refine patient selection for this therapy. Prospective trials specifically powered for mortality, incorporating individual patient data across formulations and stratifying by baseline TSAT, would meaningfully advance the field beyond the current reliance on secondary syntheses of a shared, aging trial base and would help resolve whether the modest mortality trends observed to date reflect a true, underpowered signal or the ceiling of what IV iron can achieve in this population.

## Conclusions

This umbrella review of 15 SRMAs shows that IV iron, particularly FCM, reduces HHFs and improves functional capacity and quality of life in HFrEF with ID, driven chiefly by fewer hospitalizations rather than a survival benefit, which remains unestablished. Safety appeared favorable among outcomes formally assessed, though reporting was inconsistent and hypophosphatemia was never evaluated. AMSTAR-2 appraisal found no review with high confidence; 10 were moderate, three low, and two critically low, and the apparent consistency across reviews partly reflects substantial primary-trial overlap (CCA 31.9%) rather than independent corroboration. Sex-based and TSAT-based effect modification remain hypothesis-generating. Evidence pertains predominantly to FCM in HFrEF; extrapolation to other formulations, HFmrEF, HFpEF, or acute heart failure is not supported. Dedicated trials addressing acute populations, sex differences, standardized iron-deficiency criteria, and non-FCM formulations are needed to clarify whether a true mortality benefit exists.

## Appendices

Appendix A

**Table 5:** Complete search strategy for PubMed

#	Query	Results
#1	Iron[MeSH Terms]	1,20,093
#2	Ferric Compounds[MeSH Terms]	46,472
#3	Anemia, Iron-Deficiency[MeSH Terms]	13,159
#4	(((((((((((iron deficiency[Title/Abstract]) OR (iron-deficient[Title/Abstract])) OR (intravenous iron[Title/Abstract])) OR (IV iron[Title/Abstract])) OR (parenteral iron[Title/Abstract])) OR (ferric carboxymaltose[Title/Abstract])) OR (ferinject[Title/Abstract])) OR (injectafer[Title/Abstract])) OR (ferric derisomaltose[Title/Abstract])) OR (iron isomaltoside[Title/Abstract])) OR (monofer[Title/Abstract])) OR (iron sucrose[Title/Abstract])) OR (venofer[Title/Abstract])) OR (ferumoxytol[Title/Abstract])) OR (iron dextran[Title/Abstract])	34,175
#5	#1 OR #2 OR #3 OR #4	1,80,643
#6	Heart Failure[MeSH Terms]	1,66,594
#7	(((((Heart Failure[Title/Abstract]) OR (HF[Title/Abstract])) OR (HFrEF[Title/Abstract])) OR (HFmrEF[Title/Abstract])) OR (cardiac failure[Title/Abstract])) OR (chronic heart failure[Title/Abstract])	3,16,936
#8	#6 OR #7	3,52,785
#9	#5 AND #8	2,738

Appendix B 

**Table 6:** Complete search strategy for Cochrane Library

#	Query	Results
#1	MeSH descriptor: [Iron] in all MeSH products	3,310
#2	MeSH descriptor: [Ferric Compounds] explode all trees	1,699
#3	(iron deficiency):ti,ab,kw OR (iron-deficient):ti,ab,kw OR (intravenous iron):ti,ab,kw OR (parenteral iron):ti,ab,kw OR (IV iron):ti,ab,kw	7,428
#4	(ferric carboxymaltose):ti,ab,kw OR (ferinject):ti,ab,kw OR (injectafer):ti,ab,kw OR (ferric derisomaltose):ti,ab,kw OR (iron isomaltoside):ti,ab,kw	1,044
#5	(monofer):ti,ab,kw OR (venofer):ti,ab,kw OR (iron sucrose):ti,ab,kw OR (ferumoxytol):ti,ab,kw	841
#6	(feraheme):ti,ab,kw OR (iron dextran):ti,ab,kw	227
#7	#1 OR #2 OR #3 OR #4 OR #5 OR #6	9,800
#8	MeSH descriptor: [Heart Failure] explode all trees	15,593
#9	(heart failure):ti,ab,kw OR (HF):ti,ab,kw OR (HFrEF):ti,ab,kw OR (HFpEF):ti,ab,kw OR (HFmrEF):ti,ab,kw	55,448
#10	(cardiac failure):ti,ab,kw	21,517
#11	#8 OR #9 OR #10	58,303
#12	#7 AND #11	606

Appendix C 

**Table 7:** Complete search strategy for Embase

Database	Search strategy	Records identified
Embase	('iron'/exp OR 'ferric compound'/exp OR (iron deficiency):ti,ab,kw OR (iron-deficient):ti,ab,kw OR (intravenous iron):ti,ab,kw OR (parenteral iron):ti,ab,kw OR (IV iron):ti,ab,kw OR (ferric carboxymaltose):ti,ab,kw OR (ferinject):ti,ab,kw OR (injectafer):ti,ab,kw OR (ferric derisomaltose):ti,ab,kw OR (iron isomaltoside):ti,ab,kw OR (monofer):ti,ab,kw OR (venofer):ti,ab,kw OR (iron sucrose):ti,ab,kw OR (ferumoxytol):ti,ab,kw OR (feraheme):ti,ab,kw OR (iron dextran):ti,ab,kw) AND ('heart failure'/exp OR (heart failure):ti,ab,kw OR (HF):ti,ab,kw OR (HFrEF):ti,ab,kw OR (HFpEF):ti,ab,kw OR (HFmrEF):ti,ab,kw OR (cardiac failure):ti,ab,kw)	3,754

Appendix D 

**Table 8:** Complete search strategy for Scopus

Database	Search strategy	Records identified
Scopus	TITLE-ABS-KEY("iron deficiency" OR "iron-deficient" OR "intravenous iron" OR "parenteral iron" OR "IV iron" OR "ferric carboxymaltose" OR "ferinject" OR "injectafer" OR "ferric derisomaltose" OR "iron isomaltoside" OR "monofer" OR "venofer" OR "iron sucrose" OR "ferumoxytol" OR "feraheme" OR "iron dextran") AND TITLE-ABS-KEY("heart failure" OR "HF" OR "HFrEF" OR "HFpEF" OR "HFmrEF" OR "cardiac failure")	2,856

Appendix E 

**Table 9:** Citation matrix of primary randomized controlled trials across the 15 included systematic reviews and meta-analyses A check mark (✓) indicates that the primary trial (row) was cited as an included study by the corresponding systematic review or meta-analysis (column). This matrix was used to calculate the CCA reported in the Methods and Results. Seven additional observational studies cited by individual reviews (Capone 2023, López-Vilella 2023, Arias-Barrera 2019, Jacob/AHF-ID 2020, Mistry 2019, Kaminsky 2016, and Borreda 2022) were excluded from this matrix and the CCA denominator, consistent with the Methods. CCA, corrected covered area

Primary randomized trial	Salah et al. [2]	Anker et al. [6]	Ponikowski et al. [7]	Ahmed et al. [13]	Anker et al. [14]	Awad et al. [15]	Bhatia et al. [16]	Graham et al. [17]	Martens et al. [18]	Lopez et al. [19]	Mhanna et al. [20]	Oli et al. [21]	Rangwala et al. [22]	Shalabi et al. [23]	Taha et al. [24]
Silverberg 2001						✓									
Toblli 2007	✓					✓	✓	✓	✓						
FERRIC-HF (Okonko 2008)	✓					✓	✓	✓	✓		✓	✓			
FAIR-HF (Anker 2009)	✓	✓		✓	✓	✓	✓	✓	✓		✓	✓	✓	✓	✓
IRON-HF (Beck-da-Silva 2013)						✓					✓	✓			
Toblli 2015						✓						✓			
CONFIRM-HF (Ponikowski 2015)	✓	✓	✓	✓	✓	✓	✓	✓	✓		✓	✓	✓	✓	✓
EFFECT-HF (van Veldhuisen 2017)	✓			✓		✓	✓	✓	✓		✓	✓	✓	✓	✓
Toblli 2017											✓				
EFFICACY-HF (2017)									✓						
FER-CARS-01 (2017)									✓						
ADHF RCT (Youssef 2018)										✓					
PRACTICE-ASIA-HF (Yeo 2018)	✓			✓		✓	✓	✓	✓	✓	✓	✓	✓		✓
FERRIC-HF II (Charles-Edwards 2019)						✓						✓			
AFFIRM-AHF (Ponikowski 2020)	✓	✓	✓	✓	✓	✓	✓	✓	✓	✓	✓	✓	✓	✓	
Dhoot 2020 (FCM-HF-IN)				✓		✓		✓	✓		✓	✓		✓	✓
Myocardial-IRON (Núñez 2020)						✓					✓	✓			
Akintunde 2021						✓									
IRON-CRT (Martens 2021)	✓			✓		✓		✓	✓		✓	✓	✓	✓	✓
Marcusohn 2022	✓					✓	✓		✓		✓	✓			
Caravita 2022												✓			
IRONMAN (Kalra 2022)	✓	✓			✓	✓	✓	✓	✓		✓	✓		✓	
Mollace 2022				✓											✓
HEART-FID (Mentz 2023)		✓	✓	✓		✓			✓		✓	✓	✓	✓	✓
Drozd 2024				✓											
FAIR-HF2 / DZHK05 (Anker 2025)		✓		✓										✓	
Total trials cited (of 26)	10	6	3	11	4	18	9	10	14	3	14	16	7	9	8
